# Development of blood-brain barrier-penetrating antibodies for neutralizing tick-borne encephalitis virus in the brain

**DOI:** 10.1128/msphere.00184-25

**Published:** 2025-07-07

**Authors:** Mizuki Fukuta, Sayo Fukano, Naoya Maekawa, Shintaro Kobayashi, Shunsuke Okamoto, Minato Hirano, Junko Nio-Kobayashi, Hiroaki Kariwa, Shigeru Kawakami, Satoru Konnai, Kentaro Yoshii

**Affiliations:** 1 Department of Viral Ecology, National Research Center for the Control and Prevention of Infectious Diseases, Nagasaki University 12961, Nagasaki, Japan; 2 Laboratory of Public Health, Faculty of Veterinary Medicine, Hokkaido University 12810, Hokkaido, Japan; 3 Department of Disease Control, Faculty of Veterinary Medicine, Hokkaido University 12810, Sapporo, Japan; 4 Department of Functional Glycobiology in Infectious Diseases, National Research Center for the Control and Prevention of Infectious Diseases, Nagasaki University 12961, Nagasaki, Japan; 5 Department of Pharmaceutical Informatics, Graduate School of Biomedical Sciences, Nagasaki University Graduate School of Biomedical Sciences 200674, Nagasaki, Japan; 6 Institute of Tropical Medicine (NEKKEN), Nagasaki University, Nagasaki, Japan; University of Maryland School of Medicine, Baltimore, Maryland, USA

**Keywords:** tick-borne encephalitis virus, blood-brain barrier, rabies virus glycoprotein, recombinant antibody

## Abstract

Tick-borne encephalitis virus (TBEV) belongs to the genus *Flavivirus* and causes tick-borne encephalitis (TBE), a disease characterized by severe neurological symptoms with a high mortality rate. Currently, no specific antiviral treatments have been developed for TBE. The blood-brain barrier (BBB) restricts drug delivery to the central nervous system, posing a major challenge in developing effective therapies targeting TBEV in the brain. In this study, we developed recombinant anti-TBEV antibodies fused with BBB-penetrating rabies virus glycoprotein (RVG) peptides to facilitate their transport across the BBB. The fusion of RVG peptides to the C-terminus of the heavy chain of whole antibodies or single-chain variable fragment had minimal impact on their neutralizing ability against TBEV. The RVG fusion enhanced antibody binding to the surface of a human brain endothelial cell line and increased permeability in an *in vitro* BBB model. The RVG-fused antibodies exhibited a higher transport efficiency to the brain than the non-fused antibodies following peripheral injection in mice. Notably, the peripheral administration of the RVG-fused whole antibody after viral invasion into the brain significantly neutralized TBEV in the brains of infected mice. These findings suggest that RVG-fused antibodies represent a promising therapeutic strategy for treating TBE once the virus has entered the brain.

**IMPORTANCE:**

Tick-borne encephalitis virus is a neuroinvasive pathogen that causes severe neurologic disease, significantly affecting patients' quality of life. No specific antiviral treatment is available for tick-borne encephalitis caused by virus multiplication in the brain. The delivery of drugs to the brain via peripheral administration is often obstructed by the blood-brain barrier. To develop targeted antiviral therapies for brain infections, we engineered recombinant antibodies capable of crossing the blood-brain barrier via brain-targeted ligands. These antibodies exhibited permeability across the blood-brain barrier in both *in vitro* and *in vivo* models and notably effectively neutralized the virus within the brain following peripheral administration. This study is the first to highlight the therapeutic potential of brain-targeted recombinant antibodies after viral entry into the brain, offering a promising pathway for the development of effective antiviral treatments for tick-borne encephalitis.

## INTRODUCTION

Tick-borne encephalitis virus (TBEV), which belongs to the family *Flaviviridae* and the genus *Orthoflavivirus*, is the causative agent of tick-borne encephalitis (TBE) (1). This virus is primarily transmitted by hard ticks of the genus *Ixodes* and is endemic across a wide range of the Eurasian continent (2, 3). Reports indicate that between 10,000 and 15,000 cases occur annually (2). Phylogenetic analysis has identified at least three subtypes of TBEV: Far Eastern (TBEV-FE), Siberian (TBEV-Si), and European (TBEV-EU) subtypes (4). In human cases, the severity of clinical symptoms varies depending on the TBEV subtype to which patients are exposed (5). Additionally, in mouse models, pathogenicity can vary among strains, even within the same subtype (6–8). TBEV infections are classified into two phases: the initial viremic phase and the subsequent phase characterized by neurological symptoms (5). A bite from an infected tick transmits the virus to the host’s skin (9). After initial replication in the skin, the virus is transported to the draining lymph nodes and replicates in peripheral organs, leading to the viremic phase. This phase manifests as flu-like symptoms, such as fever, headache, and fatigue (10). Subsequently, the virus invades the central nervous system (CNS) and causes neurological symptoms, including chorea and paralysis (11). Patients infected with TBEV-EU typically experience the viremic phase, whereas most patients infected with TBEV-FE present predominantly with the neurological phase, which is associated with the most severe encephalitis cases and the highest mortality rates (12, 13). Severe CNS lesions can result in death or long-term sequelae, which significantly diminish the quality of life of patients with TBE (14). Infection with TBEV-Si has also been reported to lead to persistent infection, resulting in chronic TBE (15). However, there is currently no approved treatment for TBE, and only supportive care is provided to patients with encephalitis.

Previous studies have shown that anti-TBEV monoclonal antibodies exhibit protective efficacy in TBEV-infected mice when administered peripherally during the viremic phase (16, 17). However, no evidence suggests that these antibodies remain effective once the virus has invaded the brain. Additionally, no studies have developed brain-targeted drug delivery systems (DDSs) for anti-TBEV molecules.

The blood-brain barrier (BBB) is a highly selective biological barrier that separates blood vessels from brain parenchyma. It is composed of microvascular endothelial cells and tight junctions, playing a crucial role in preventing harmful agents and microorganisms from entering the CNS via the bloodstream (18). However, this barrier also limits the effective delivery of drugs into the CNS, depending on factors, such as molecular size and hydrophilicity (19). Therefore, it is critical to transport anti-TBEV molecules to the brain beyond the BBB to develop TBE therapeutics.

The BBB has several endogenous transport pathways for delivering nutrients and metabolites to the CNS, with receptor-mediated transcytosis (RMT) being one of its primary routes (20). In RMT, ligands bind to receptors on the surface of the brain endothelial cells on the blood vessel side, are transported across the cell via vesicle trafficking, and are released into the brain parenchyma. Recently, peptides from these ligands, which are responsible for BBB penetration, have been used for effective drug delivery into the CNS (21).

A short peptide derived from rabies virus glycoprotein (RVG) has been investigated as a potential candidate for a brain-targeted DDS. RVG comprises 29 amino acids that bind to the nicotinic acetylcholine receptor (nAChR) (22). Kumar et al. (23) reported that RVG-conjugated small interfering RNA targeting the Japanese encephalitis virus was successfully delivered to the brain and showed therapeutic effects in mice inoculated with the virus.

In this study, we engineered anti-TBEV recombinant antibodies fused to RVG and evaluated their neutralizing abilities, as well as their ability to cross the BBB, both *in vitro* and *in vivo*. We further assessed the therapeutic efficacy of these antibodies through peripheral administration in TBEV-infected mice. Our findings indicate that RVG-fused antibodies are promising candidates for effectively targeting TBEV in the brain.

## MATERIALS AND METHODS

### Cells and viruses

Baby hamster kidney (BHK-21) cells were cultured in Eagle’s Minimum Essential Medium (Fujifilm, Tokyo, Japan) supplemented with 8% heat-inactivated fetal bovine serum (FBS). Human brain capillary endothelial cells (hCMEC/D3) purchased from Merck were cultured in an EGM-2 Endothelial Cell Growth Medium-2 Bullet Kit (Lonza, Verviers, Belgium) supplemented with 10% heat-inactivated FBS. The cells were grown in flasks coated with rat tail collagen type I at a concentration of 5 µg/cm^2^ (Corning, New York).

The recombinant virus from an infectious clone of the TBEV Oshima 5-10 strain was generated as previously described (24).

### Construction of plasmids encoding recombinant antibodies

To obtain the sequences for the variable regions of monoclonal IgG1 19/1786, total RNA was isolated from 19/1786 murine hybridoma cells kindly provided by Dr. Daniel Ruzek (Masaryk University) (25) and reverse-transcribed using SuperScript II Reverse Transcriptase (Life Technologies, Carlsbad, CA). The full-length cDNA sequences of the variable regions of the heavy (V_H_) and light (V_L_) chains were determined using the rapid amplification of the cDNA end technique (26). The V_H_ and V_L_ sequences of 19/1786 are available in publicly accessible databases, with NCBI GI numbers 1345621741 and 1345621743, respectively. The V_H_ and V_L_ sequences of T025 derived from a patient with TBE were obtained from a previous study (27) (NCBI GI numbers: 2023403196 and 2023403197, respectively). The sequences of the constant regions of murine IgG1, including the constant regions of heavy (C_H_) and light (C_L_) chains, were obtained from publicly available databases.

**Fig 1:**
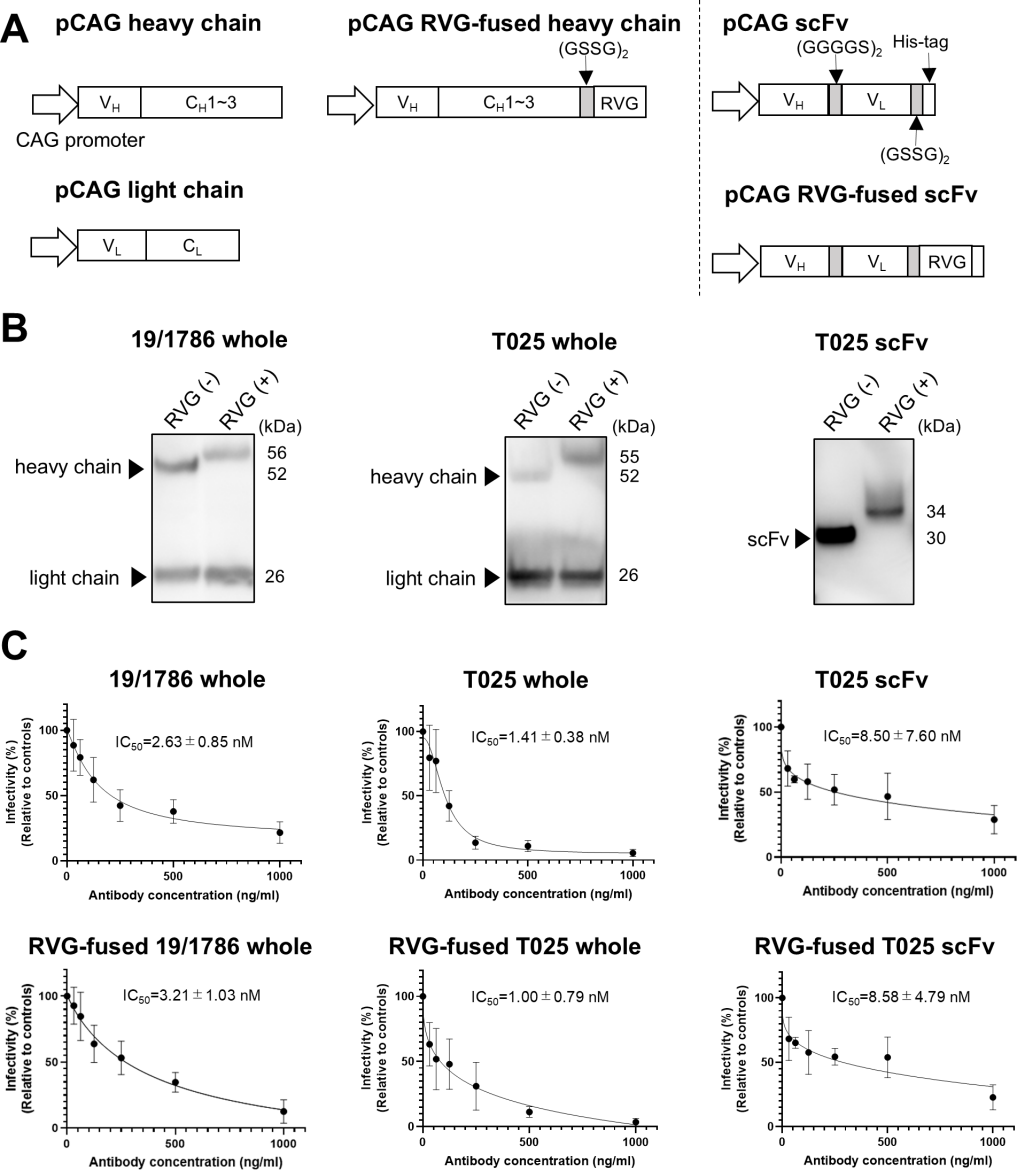
Expression of recombinant antibodies and their neutralizing abilities against tick-borne encephalitis virus (TBEV). (**A**) Schematic representation of plasmids encoding the heavy and light chains of rabies virus glycoprotein (RVG)-fused 19/1786 or T025 and RVG-fused T025 scFv. The glycine/serine (GS) linker and RVG were fused with the 3′ end of the heavy chain of each antibody. The GS linker, RVG, and His-tag were fused with the 3′ end of the scFv. V_H_, variable region of the heavy chain; C_H_, constant region of the heavy chain; V_L_, variable region of the light chain; C_L_, constant region of the light chain; GS linker, polypeptide linkers composed of G and S. (**B**) Expression and secretion of recombinant antibodies. Expi293F cells were transfected with plasmids expressing the heavy and light chains of each antibody or T025 scFv. Supernatants were collected 2 days post-transfection and analyzed by western blotting. The heavy and light chains and scFv were detected using anti-mouse IgG horseradish peroxidase (HRP) or anti-His-tag HRP. RVG (−): RVG-non-fused; RVG (+): RVG-fused. (**C**) Neutralizing abilities of recombinant antibodies. The neutralizing ability of each recombinant antibody was assessed by PRNT using the TBEV Oshima 5-10 strain. The half-maximal inhibitory concentration (IC_50_) of each antibody is presented as the mean ± standard deviation (SD) of quadruplicates.

The DNA sequences encoding the 19/1786 and T025 antibodies were synthesized by Integrated DNA Technologies, Inc. (IA). To construct DNA fragments encoding the heavy and light chains of 19/1786 and T025, V_H_ and V_L_ fragments were fused to the C_H_ and C_L_ regions of murine IgG1, respectively, using overlap PCR with PrimeSTAR MAX DNA Polymerase (Takara Bio, Shiga, Japan). To construct DNA fragments encoding the T025 single-chain variable fragment (scFv), gene fragments of V_H_ and V_L_ were fused using flexible polypeptide linkers composed of glycine (G) and serine (S) (GGGGS). Additionally, a His-tag was appended to the 3′ end of the DNA construct. DNA fragments encoding RVG (amino acid sequence: YTIWMPENPRPGTPCDIFTNSRGKRASNG) were fused to the 3′ end of the C_H_ or V_L_ of scFv using GS linkers (GSSG) using overlap PCR (Fig. 1A). The obtained gene fragments were inserted into EcoRV/XhoI-digested pCAGGS plasmids using the In-Fusion HD Cloning Kit (Takara Bio).

### Expression and purification of recombinant antibodies

For the expression of whole recombinant antibodies, both plasmids encoding the RVG-fused heavy and light chains of 19/1786 or T025 were transfected into Expi293F cells (Life Technologies) using Expifectamine 293 reagent (Life Technologies) following the manufacturer’s instructions. To express T025 scFv, a plasmid encoding the RVG-fused T025 scFv was transfected.

The cells were cultured in Expi293 Expression Medium (Life Technologies) for 2 to 7 days. The supernatants were collected and filtered through 0.22 µm membrane filters (Merck, Darmstadt, Germany). The whole antibodies in the supernatant were purified using AbCapture Extra (Protenova, Kagawa, Japan). The scFv antibodies were purified using the TALON Metal Affinity Resin (Takara Bio). The elution buffer used for purification was exchanged with phosphate-buffered saline (PBS) using PD Mini Trap G-25 (Cytiva, Tokyo, Japan).

### SDS-PAGE and western blotting

Proteins in the supernatants of transfected cells were separated by SDS-PAGE using an EHR-T520L e-PAGEL HR (ATTO, Tokyo, Japan) and transferred to a polyvinylidene difluoride membrane (Merck). The membrane was blocked with Block Ace (KAC, Kyoto, Japan) and incubated with horseradish peroxidase (HRP)-conjugated goat anti-mouse IgG antibody (Jackson ImmunoResearch, West Grove, PA) for whole antibody detection or HRP-conjugated mouse anti-His tag antibody (MBL, Tokyo, Japan) for scFv detection. The proteins were visualized using a chemiluminescent HRP substrate (Merck) and LuminoGraph I (ATTO).

### Neutralization assay

Serial dilutions of recombinant antibodies were mixed with an equal volume of TBEV Oshima 5-10 suspension and incubated at 37°C for 1 h. The virus-antibody complex was then inoculated into BHK cells seeded in a 12-well plate and incubated at 37°C for 1 h. Following incubation, an overlay medium containing 1.5% carboxymethylcellulose (Fujifilm) and modified Eagle’s medium (Life Technologies) was supplemented with 2% heat-inactivated FBS. The absolute IC_50_ (half-maximal inhibitory concentrations) values were determined using nonlinear regression analysis.

### Immunofluorescence assay

An immunofluorescence assay (IFA) was performed to evaluate the binding ability of the RVG-fused antibodies to the surface of hCMEC/D3 cells. The cells were seeded on eight-well chamber slides (ibidi, Bayern, Germany) and incubated at 37°C with 5% CO_2_ until they reached 70–80% confluency. The cells were then fixed with 4% paraformaldehyde and blocked with PBS containing 2% bovine serum albumin. Subsequently, 10 µg of each recombinant antibody was added per well and incubated at 4°C for 4 h, followed by staining with DAPI and Alexa Fluor 594-conjugated goat anti-mouse IgG (Life Technologies) or Alexa Fluor 488-conjugated mouse anti-His tag antibody (MBL). Images were acquired using an FV3000 Confocal Laser Scanning Microscope (Evident, Tokyo, Japan) and analyzed with FV31S-SW software (Evident).

### Permeability assay of recombinant antibodies using an *in vitro* BBB model

To construct the *in vitro* BBB model, 12-well Transwell inserts (Corning) were coated with type I collagen, and hCMEC/D3 cells were seeded at a density of 2 × 10^5^ cells/cm^2^. The transepithelial electrical resistance (TEER) was monitored using a Millicell ERS-2 (Merck). TEER values were calculated as follows:

TEER (Ω·cm^2^) = (*R*_cell monolayer_ − *R*_blank_) × area of the membrane (cm^2^),

where *R*_cell monolayer_ represents the resistance of the cell monolayer on the insert and *R*_blank_ represents the resistance of the insert without cells.

The permeability assay was conducted only when the TEER value of the cell monolayer exceeded 20 Ω·cm^2^ (28). In this study, the TEER of this model was consistent with previously reported values (30–40 Ω·cm2) (29). To confirm the barrier function of the *in vitro* BBB model, the transport ratio was evaluated using fluorescein isothiocyanate (FITC)-dextran (Merck) with 4,000 and 70,000 Da molecular weights (MW). A total of 300 pmol of each FITC-dextran was added to the upper chamber with or without cell monolayers and incubated for 3 h at 37°C. After incubation, the fluorescence intensity of FITC-dextran transported into the lower chamber was measured using SpectraMax iD5 (Molecular Devices, Sunnyvale, CA).

To evaluate the transport of recombinant antibodies using the *in vitro* BBB model, a total of 300 pmol of each recombinant antibody was added to the upper chamber coated with hCMEC/D3 and incubated for 3 h at 37°C. After incubation, the antibodies transported into the lower chamber were detected using an enzyme-linked immunosorbent assay (ELISA), as previously described (30). The total amount of antibody or FITC dextran in the lower chamber was determined by interpolating the sample optical density (OD) or fluorescence value against a calibration curve generated from known concentrations of antibodies or FITC dextran. The transport ratio was calculated as follows:

Transport ratio (%) = (total amount of antibody or FITC dextran detected in the lower chamber in moles) / (amount of antibody or FITC dextran added to the upper chamber in moles) × 100.

To evaluate viral neutralization by the transported antibodies in the *in vitro* BBB model, 1,000 PFU of TBEV Oshima 5-10 was added to the lower chamber of the model. Simultaneously, 300 pmol of each antibody was added to the upper chamber and incubated for 3 h. After incubation, the viral titer in the lower chamber was evaluated using a plaque assay.

### Evaluation of recombinant antibody transport to the brain in a mouse model

Female BALB/c mice were purchased from Japan SLC, Inc. (Shizuoka, Japan). The recombinant antibodies were biotinylated using a Biotinylation Kit (Sulfo-OSu) (Dojindo, Kumamoto, Japan) following the manufacturer’s instructions. A total of 1.2 nmol of the biotinylated recombinant antibodies or PBS was injected intraperitoneally into the mice. After 24 h, the brains were collected, homogenized in Eagle’s medium containing 2% FBS, and centrifuged at 6,000 rpm for 10 min at 4°C. The resulting supernatants were subjected to ELISA to detect biotinylated antibodies in the brain. For the ELISA, 96-well plates were coated overnight at 4°C with goat anti-mouse IgG (Jackson ImmunoResearch) or rabbit anti-His tag antibody (Jackson ImmunoResearch) and blocked with Block Ace (KAC) for 30 min at 37°C. After washing with PBS supplemented with 0.05% Tween 20 (PBS-T), the plates were incubated with serially diluted samples for 1 h at 37°C. After washing with PBS-T, the plates were incubated with StrepTactin-HRP conjugates (Bio-Rad, Hercules, CA) for 1 h at 37°C. After washing, the StrepTactin-HRP reaction was developed using OPD, and after a 30 min incubation, the OD value was measured using SpectraMax iD5 (Molecular Devices). The total amount of antibodies accumulated in the brain was determined by interpolating the OD values against a calibration curve generated from known concentrations of the antibodies. The percent of injected dose per gram brain (%ID/g brain) was calculated as follows:

%ID/g brain = (amount of accumulated antibody in 1 g of brain tissue) / (total amount of antibody injected peripherally) × 100.

To evaluate viral neutralization in the brain following the peripheral administration of recombinant antibodies, 1,000 PFU of TBEV Oshima 5-10 was inoculated subcutaneously into each mouse. From days 4–5 post-infection, a total of 0.3 nmol of RVG-fused antibodies was injected intraperitoneally for three consecutive days. On day 7 post-infection, the brains were collected and homogenized, as described above. Viral titers in the brain were evaluated using a plaque assay.

## RESULTS

### Development of RVG-fused anti-TBEV recombinant antibodies

In this study, we explored the use of anti-TBEV recombinant antibodies as antiviral molecules capable of neutralizing the virus in the brain by fusing them with RVG, a known BBB-penetrating peptide. Two monoclonal antibodies capable of neutralizing multiple strains of TBEV were used to develop the recombinant antibodies. The 19/1786 antibody was derived from murine hybridoma cells that produce IgG1 (31), while T025 originated from a patient with TBE (27). The whole chimeric T025 recombinant antibody was developed by fusing the variable regions of T025 to the C_H_ and C_L_ chains of murine IgG1. Additionally, the scFvs derived from 19/1786 and T025 were engineered by linking their V_H_ and V_L_ chains using the GS linkers. To enable these recombinant antibodies to cross the BBB, the BBB-penetrating peptide RVG, which binds to nAChRs, was fused to the C-terminus of the heavy chain of the whole antibody or T025 scFv (Fig. 1A). The scFv derived from 19/1786 exhibited no neutralizing ability and was therefore excluded from further experiments.

Recombinant antibodies were expressed in Expi293F cells and analyzed via western blotting (Fig. 1B). The expected band sizes for each chain, including the increased molecular weight due to RVG fusion, were observed in the culture supernatant. Purified recombinant antibodies from the supernatant were used for subsequent analysis.

To assess whether RVG fusion affected the neutralizing ability of the recombinant antibodies, a plaque reduction neutralization test (PRNT) was performed using the TBEV Oshima 5-10 strain. Although the scFv antibodies exhibited a relatively lower neutralizing ability than the whole antibodies, the RVG-fused antibodies exhibited a neutralizing ability comparable to that of their non-fused counterparts (Fig. 1C). These results indicate that RVG fusion had minimal impact on the neutralizing efficacy of the recombinant antibodies.

### Binding of RVG-fused antibodies to the cell surface of brain capillary endothelial cells

**Fig 2:**
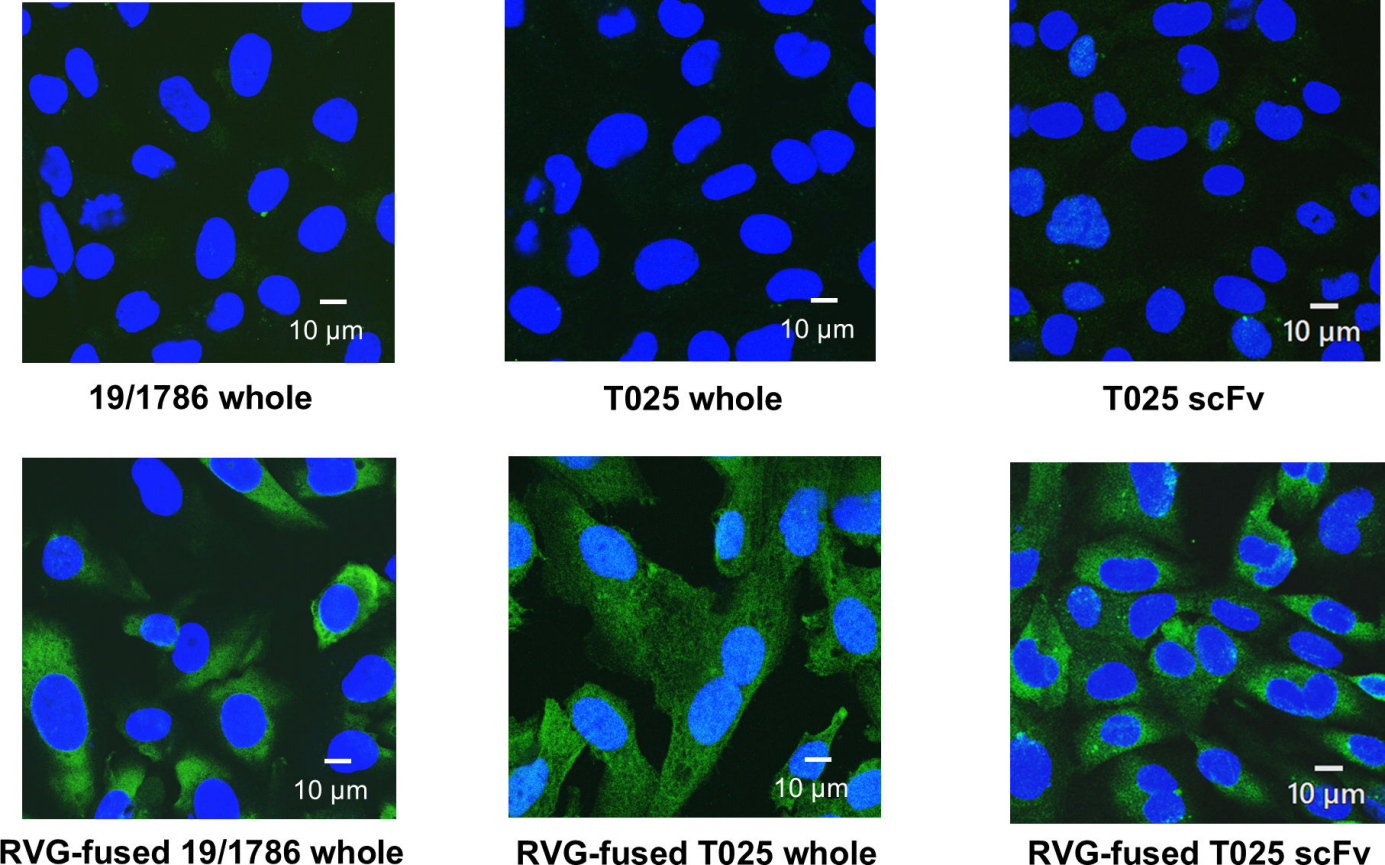
Binding abilities of RVG-fused recombinant antibodies to the cell surface of hCMEC/D3 cells. hCMEC/D3 cells were fixed with paraformaldehyde and incubated with each antibody at 4°C for 4 h. Antibodies bound to the cell surface were detected using an Alexa Fluor-conjugated goat anti-mouse IgG or mouse anti-His tag antibody (green), and nuclei were stained with DAPI (blue).

The RVG peptide has been reported to bind to nAChRs (22). Previous studies have shown that this binding on brain capillary endothelial cells triggers RMT (20). To evaluate the binding of RVG-fused antibodies, IFA was performed using paraformaldehyde-fixed hCMEC/D3 cells without permeabilization, an established human brain capillary endothelial cell line. hCMEC/D3 cells treated with each RVG-fused antibody exhibited strong cell surface fluorescence, whereas no fluorescence was observed when the cells were treated with antibodies lacking the RVG fusion (Fig. 2). These results indicate that RVG fusion enabled the recombinant antibodies to bind to the cell surface, suggesting that the antibodies interacted with the cell surface receptor via the RVG peptide.

### Evaluation of RVG-fused recombinant antibodies *in vitro*

**Fig 3:**
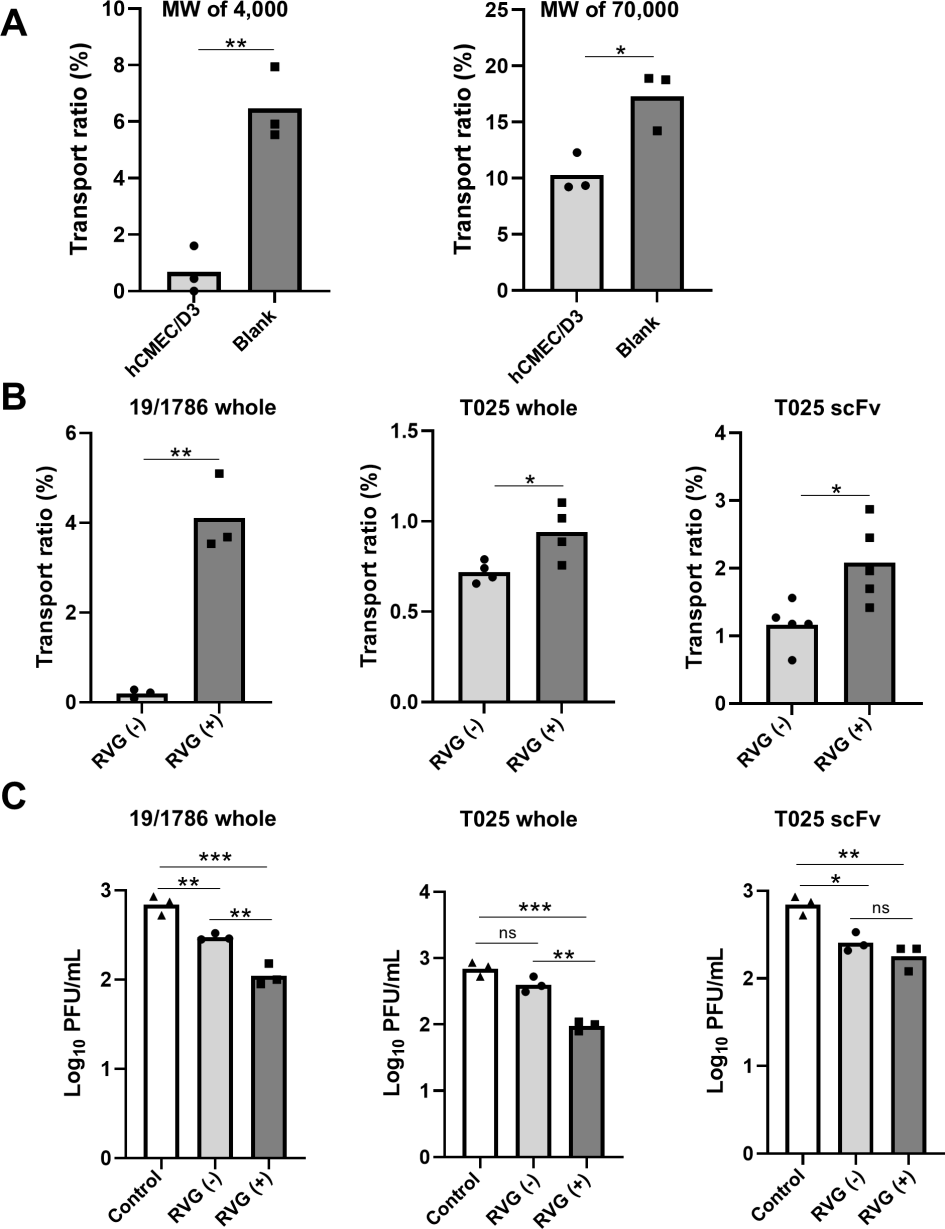
Evaluation of the blood-brain barrier (BBB) permeability of recombinant antibodies *in vitro*. (**A**) Transport ratio of fluorescein isothiocyanate (FITC)-dextran across the *in vitro* BBB model. FITC-dextran was added to the upper chamber with or without hCMEC/D3 cells. After 3 h, the medium in the lower chamber was collected, and fluorescence was measured. Data are presented as the mean ± SD from triplicates. Statistical analyses were performed using an unpaired Student’s *t*-test (*P  <  0.05, **P  <  0.005). (**B**) Transport ratio of recombinant antibodies across the *in vitro* BBB model. The antibodies were added to the upper chamber coated with hCMEC/D3 cells. After 3 h, the transported antibodies in the lower chamber were analyzed using ELISA. Data are presented as the mean ± SD from three, four, or five replicates. Statistical analyses were performed using an unpaired Student’s *t*-test (*P  <  0.05, **P  <  0.005). RVG (−): RVG-non-fused; RVG (+): RVG-fused. (**C**) Viral titers in the lower chamber of the *in vitro* BBB model. Each antibody was added to the upper chamber coated with hCMEC/D3 cells, whereas the lower chamber contained TBEV Oshima 5-10. After 3 h, viral titers in the lower chamber were measured using a plaque assay. Data are presented as the mean ± SD from triplicates. Statistical analyses were performed using Tukey’s multiple-comparisons test (*P  <  0.05, **P  <  0.005, ***P  <  0.0001, ns: not significant). Control: no antibody treatment; RVG (−): RVG-non-fused; RVG (+): RVG-fused.

We evaluated the BBB permeability of the RVG-fused antibodies using an *in vitro* BBB model with hCMEC/D3 cells, an established laboratory model to simulate the BBB (32). The barrier function of this model was evaluated using FITC-dextran (MW 4,000 and 70,000), a marker for BBB permeability, by measuring the fluorescent intensity of FITC transported to the lower chamber. The transport ratio of both dextran molecules across the cell monolayer was significantly lower than that in the blank wells lacking a cell monolayer (Fig. 3A). These results indicated that the barrier was functionally effective in this model.

Next, the BBB permeability of the recombinant antibodies was evaluated using this *in vitro* BBB model. Each antibody was added to the upper chamber seeded with hCMEC/D3 cells, and antibodies transported to the lower chamber were quantified using ELISA. The transport ratios (%) of the RVG-fused antibodies were significantly higher than those of the non-fused antibodies (Fig. 3B), indicating that RVG fusion increased the permeability of the recombinant antibodies *in vitro*.

The neutralizing ability of RVG-fused antibodies that crossed the hCMEC/D3 cell monolayer was evaluated using the model. TBEV was introduced into the lower chamber, followed by the addition of each antibody. Viral titers in the lower chamber after 3 h were quantified using plaque assay. The viral titers in samples treated with RVG-fused whole antibodies were significantly lower than those in the control group and those treated with non-fused antibodies for both 19/1786 and T025 (Fig. 3C). These results indicate that the RVG-fused whole antibodies successfully crossed the cell monolayer and neutralized TBEV in the lower chamber. However, no significant differences in viral titers were observed between treatments with RVG-fused and non-fused T025 scFv.

### *In vivo* brain targeting and therapeutic efficacy of RVG-fused antibodies

**Fig 4:**
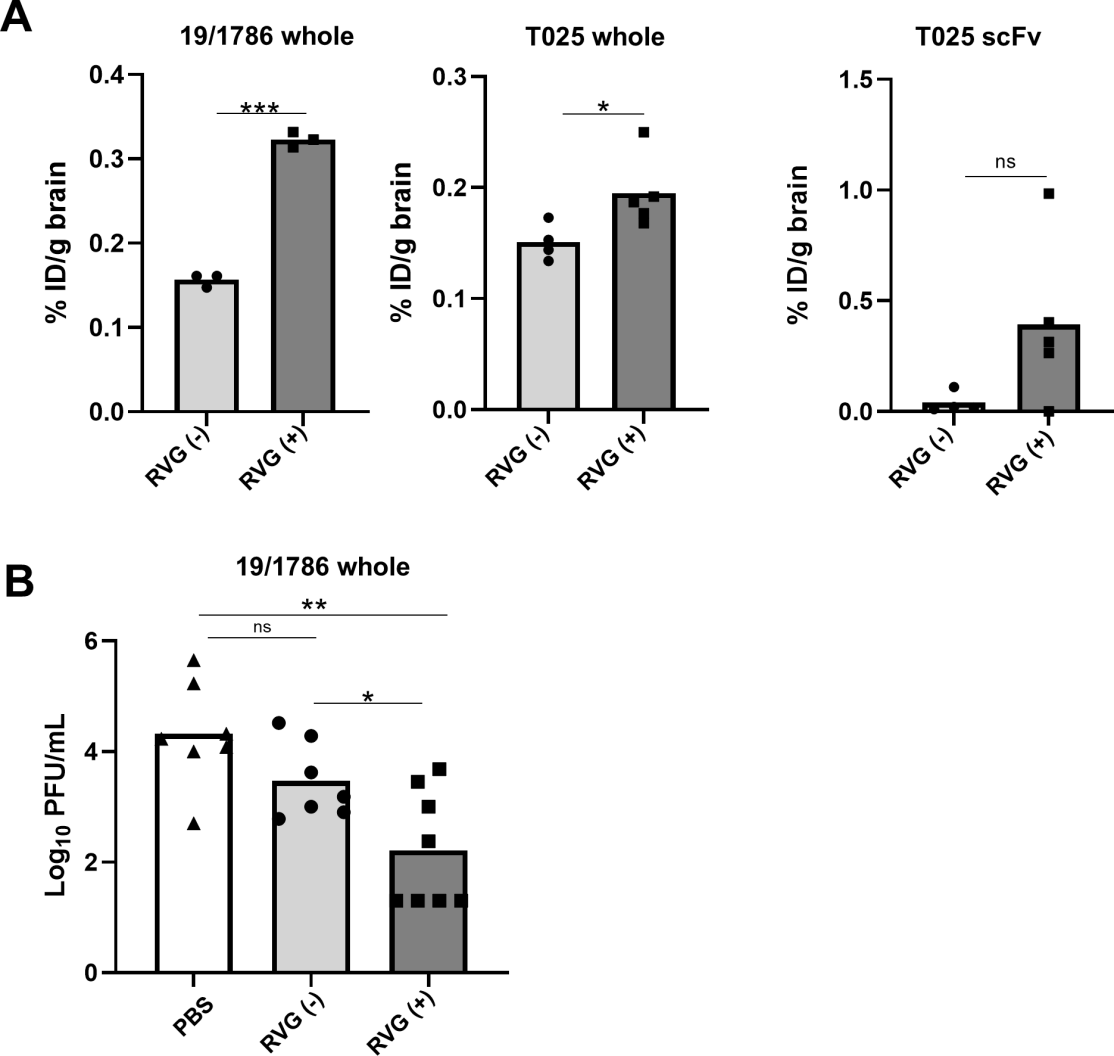
BBB permeability and therapeutic efficacy of RVG-fused antibodies *in vivo*. (**A**) Transport of RVG-fused antibodies into the mouse brain after peripheral administration. Mice were injected intraperitoneally with 1.2 nmol biotinylated antibodies. After 24 h, the brains were collected, and the antibody concentrations were measured. Data are presented as the mean ± SD from three, four, or five replicates. Statistical analyses were performed using an unpaired Student’s *t*-test (*P  <  0.05, ***P  <  0.0001, ns: not significant). RVG (−): RVG-non-fused; RVG (+): RVG-fused. (**B**) Inhibition of viral replication in the mouse brain following the administration of RVG-fused antibodies. Mice infected with TBEV Oshima 5-10 were treated intraperitoneally with RVG-fused antibodies for three consecutive days. On day 7 post-infection, the brains were collected, and the viral titers were measured. Data are presented as the mean ± SD from seven or eight replicates. Statistical analyses were performed using Tukey’s multiple-comparisons test (*P  <  0.05, **P  <  0.005, ns: not significant). RVG (−): RVG-non-fused; RVG (+): RVG-fused.

Previous studies have reported that peripherally administered RVG-conjugated siRNAs can reach the mouse brain (23). To evaluate whether RVG-fused recombinant antibodies can penetrate the brain *in vivo*, their accumulation in the brain was quantified. Each biotinylated antibody was administered intraperitoneally to mice, and 24 h post-injection, the concentration of the antibodies in the brain tissue was measured using ELISA. The %ID/g brain value in mice treated with RVG-fused whole antibodies was significantly higher than that in mice treated with non-fused whole antibodies (Fig. 4A). This result indicates that the RVG-fused antibodies successfully crossed the BBB *in vivo*. Although one mouse treated with RVG-fused T025 scFv showed undetectable antibody levels, the %ID/g brain in the remaining mice was higher than that in the mice treated with non-fused antibodies. Further evaluation of the therapeutic efficacy of the RVG-fused antibodies in TBEV-infected mice was performed using the RVG-fused 19/1786 whole antibody.

To assess the protective effects of RVG-fused antibodies against TBEV infection *in vivo*, mice were subcutaneously inoculated with TBEV. On days 4–6 post-infection, when the virus had invaded the brain and was undetectable in the peripheral tissues (33), RVG-fused antibodies were intraperitoneally administered. On day 7 post-infection, the brains were collected, and the viral titers in the brain tissue were measured using a plaque assay. Viral titers were significantly lower in mice treated with RVG-fused antibodies than in mice who received non-fused antibodies and PBS (Fig. 4B). These results indicate that the RVG-fused antibodies effectively crossed the BBB and neutralized TBEV in the brain.

## DISCUSSION

In this study, we developed recombinant antibodies against TBEV fused with RVG to target and neutralize TBEV in the brain. The RVG-fused antibodies neutralized TBEV as effectively as non-fused antibodies. These RVG-fused antibodies crossed the endothelial cell monolayer in an *in vitro* BBB model and were transported to the mouse brain following peripheral administration. Furthermore, the peripheral administration of these antibodies significantly neutralized TBEV in the brain.

The fusion of RVG peptides had minimal impact on antibody neutralization. RVG is a well-studied brain targeting ligand. Previous studies have shown that the conjugation or fusion of ligands with antibodies can affect their binding capabilities. For instance, the fusion of antibodies targeting the TfR with antibodies against amyloid beta increased their binding affinities (34), whereas the conjugation of angiopep-2 to antibodies targeting the epidermal growth factor receptor reduced their binding affinities (35). Phoolcharoen et al. (36) reported that the RVG fusion at the C-terminus did not impair the neutralizing ability of scFv against rabies virus, which is consistent with our findings.

The conversion of the 19/1786 and T025 antibodies into scFv forms resulted in reduced neutralizing capabilities compared to their whole counterparts. The Fab region of 19/1786 binds to domain III of the envelope (E) protein of TBEV while simultaneously binding to either domain I or II of the E protein (31). Similarly, T025 recognizes an epitope analogous to that of 19/1786 (27). It is possible that converting whole antibodies into scFvs leads to a loss of epitope recognition owing to alterations in the number of antigen-binding sites or changes in their three-dimensional conformation. Additionally, the length of the GS linker and the arrangement of the V_H_ and V_L_ peptides influence the binding affinity of scFv, as reported in previous studies (37, 38). Based on these findings, further optimization of scFv design could enhance its neutralization potential.

RVG-fused antibodies demonstrated the ability to cross the BBB. Multiple molecular transport pathways exist at the BBB. Small lipid-soluble molecules (<500 Da) can cross the BBB via passive diffusion (39, 40). In adsorptive-mediated transcytosis, positively charged molecules interact with negatively charged cell membranes of brain capillary endothelial cells, allowing them to cross the BBB through adsorptive endocytosis (39). Among them, RMT is the primary pathway for large non-cationic proteins, such as antibodies and nanoparticles (20). Our *in vitro* studies demonstrated that RVG-fused antibodies bound to the surface of hCMEC/D3 cells, as indicated by the IFA results. It has been reported that RVG binds to the nAChR (22), which is expressed on hCMEC/D3 cells (36). The RVG-fused antibodies successfully crossed the hCMEC/D3 cell monolayer in an *in vitro* BBB model, which has been utilized to study the BBB-crossing capabilities of large molecules, such as RVG-conjugated antibodies and nanoparticles, anti-TfR antibodies, and angiopep-2 conjugates (34, 41, 42). The permeability of RVG-fused antibodies *in vitro* was comparable to that reported in previous studies evaluating angiopep-2-conjugated nanocapsules or RVG-conjugated nanoparticles and liposomes using *in vitro* BBB models (41–44). In *in vivo* studies, peripherally administered RVG-fused antibodies reached the mouse brain, consistent with prior studies using RVG-conjugated nanoparticles and liposomes (41, 43, 45). Collectively, these findings suggest that our RVG-fused antibodies cross the BBB via RMT by binding to nAChRs, enabling their entry into the brain. Previous studies have shown that TBEV multiplication in the CNS increases BBB permeability (46, 47), which may allow antibodies without RVG to enter the CNS (48). However, it remains unclear whether antibody-based therapies can exert therapeutic effects during the period of BBB disruption. A slight increase was observed in the transportation of the human-derived T025 antibody fused with the RVG peptide into the brains of mice, as compared to the mouse-derived 19/1786. Generally, antibodies from other species trigger an immune response, leading to degradation of the antibodies and potential adverse effects (49, 50). Several Food and Drug Administration-approved humanized antibodies targeting neurodegenerative diseases have been shown to cause relatively mild side effects in clinical studies (51, 52). A similar safety profile may be expected for our BBB-penetrating antibody. Therefore, the clinical application of human-derived or humanized antibodies should be examined to ensure safer and more effective treatment strategies (53, 54).

Viral neutralization in the brain by RVG-fused antibodies was assessed using an *in vitro* BBB model. RVG-fused whole antibodies that crossed the *in vitro* BBB model effectively neutralized the virus, whereas non-fused antibodies exhibited a lower neutralizing activity. The reduced neutralizing capability of the T025 scFv, compared to whole antibodies, may explain why no significant differences in viral titers were observed between RVG-fused and non-fused T025 scFv despite the higher transport ratio of the RVG-fused antibody.

RVG-fused antibodies administered intraperitoneally effectively neutralized the viruses in the brains of TBEV-infected mice. These antibodies that crossed the BBB are considered to neutralize the virus released from neuronal cells in the brain. It is also possible that RVG-fused antibodies were internalized into neuronal cells and affected the virus within the cells (36, 44). However, this is unlikely, given the limited accessibility of antibodies to TBEV virions, which are enclosed within intracellular membranes, such as the ER (55). Previous studies have demonstrated that viral titers in the brain correlate with the severity of histopathological changes, including significant neuronal degradation and inflammatory responses, in TBEV-infected mice (56). Similar pathological findings in the brain have been observed in fatal human TBE cases (57). Comparable therapeutic effects have also been reported in lethal mouse models of other encephalitic flavivirus infections, where antiviral treatment reduced viral titers in the brain (58, 59). Based on these findings, RVG-fused antibodies are considered to possess therapeutic potential by neutralizing TBEV within the brain.

The reduction of the virus in the brain was successfully achieved through the peripheral administration of RVG-fused antibodies after virus entered the brain. Most *in vivo* studies administered antivirals peripherally during the early phase of infection, when the virus primarily circulates in the peripheral blood (16, 17, 60, 61). In contrast, in this study, antibodies were administered after the viral invasion of the brain. This is the first study to demonstrate the therapeutic potential of an antiviral treatment for TBE in the later stages of infection. However, the effectiveness of RVG-fused antibodies during the acute neurological phase remains to be verified. Further studies are needed to optimize the antibody dosage and administration frequency in order to achieve therapeutic efficacy on survival and clinical manifestations in TBEV-infected mice. Nonetheless, these BBB-penetrating antivirals could potentially exhibit therapeutic effects by preventing neurological disease when administered during the febrile phase, which is presumably associated with infections by the TBEV-EU (2). Furthermore, after the acute encephalitis phase, persistent TBEV infections have been linked to an increase in focal CNS lesions, leading to chronic neurological symptoms and long-term sequelae (46, 62, 63). RVG-fused antibodies may also have the potential to mitigate chronic TBE by neutralizing TBEV during persistent infection.

A combination of BBB-crossing molecules targeting both intra- and extracellular viruses could enhance treatment efficacy for TBEV within the brain. Small antiviral molecules, such as nucleoside analogs and siRNAs, have been reported to inhibit TBEV replication in infected cells (64, 65). RVG-conjugated small antiviral molecules, such as siRNAs, are assumed to cross the BBB via RMT and are subsequently internalized into neuronal cells in the brain via receptor-mediated endocytosis following RVG binding to nAChRs on the neuronal cell surface (23, 66). Kumar et al. (23) previously demonstrated that siRNAs conjugated to RVG peptides crossed the BBB and were internalized in neurons and provided therapeutic effects in JEV-infected mice. A synergistic therapeutic strategy by the co-delivery of antibodies and small molecules, such as the combination of RVG-fused antibodies with siRNAs, could target both intra- and extracellular viruses.

In summary, we have demonstrated, for the first time, the therapeutic potential of BBB-penetrating RVG-fused antibodies after viral entry into the brain. This brain-targeted DDS utilizing RVG peptides holds potential for applications in the treatment of other CNS diseases and in advancing research on neurophysiological functions of the brain. Additionally, combining RVG-fused antibodies with antiviral molecules targeting intracellular viruses is expected to enhance therapeutic efficacy. Future optimization of the RVG-fused molecule design and administration strategies will pave the way for effective antiviral treatments for TBE.
